# Wearable Neurophysiological Recordings in Middle-School Classroom Correlate With Students’ Academic Performance

**DOI:** 10.3389/fnhum.2018.00457

**Published:** 2018-11-12

**Authors:** Yu Zhang, Fei Qin, Bo Liu, Xuan Qi, Yingying Zhao, Dan Zhang

**Affiliations:** ^1^Institute of Education, Tsinghua University, Beijing, China; ^2^Department of Psychology, School of Social Sciences, Tsinghua University, Beijing, China

**Keywords:** wearable neurophysiology recordings, skin conductance, ambulatory assessment, middle school, academic performance

## Abstract

The rapid development of wearable bio-sensing techniques has made it possible to continuously record neurophysiological signals in naturalistic scenarios such as the classroom. The present study aims to explore the neurophysiological correlates of middle-school students’ academic performance. The electrodermal signals (EDAs) and heart rates (HRs) were collected via wristband from 100 Grade seven students during their daily Chinese and math classes for 10 days in 2 weeks. Significant correlations were found between the academic performance as reflected by the students’ final exam scores and the EDA responses. Further regression analyses revealed significant prediction of the academic performance mainly by the transient EDA responses (*R*^2^ = 0.083, *p* < 0.05, with Chinese classes only; *R*^2^ = 0.030, *p* < 0.05, with both Chinese and math classes included). By combining the self-report data about session-based general statuses and the neurophysiological data, the explained powers of the regression models were further improved (*R*^2^ = 0.095, *p* < 0.05, with Chinese classes only; *R*^2^ = 0.057, *p* < 0.05, with both Chinese and math classes included), and the neurophysiological data were shown to have independent contributions to the regression models. In addition, the regression models became non-significant by exchanging the academic performances of the Chinese and math classes as the dependent variables, suggesting at least partly distinct neurophysiological responses for the two types of classes. Our findings provide evidences supporting the feasibility of predicting educational outputs by wearable neurophysiological recordings.

## Introduction

The rapid development of wearable bio-sensing techniques has made it possible to continuously record neurophysiological signals in naturalistic scenarios, such as driving, gaming, studying, and, etc. (Rutherford, 2010; Jacucci et al., 2015). The wearable bio-sensing devices, usually taking the form of a wristband, a chestband, or a headband, are capable of recording human neurophysiological signals without interrupting the participants’ performance. As human neurophysiological signals have long been acknowledged to be effective indicators of a variety of cognitive functions such as attention, engagement, emotion, and, etc. (Driver, 2001; Picard et al., 2001; Li et al., 2015; Cowley et al., 2016), these wearable recordings are expected to provide a real-time, objective measurement of human cognitive statuses in real-world situations beyond the laboratory.

The application of wearable neurophysiological recordings in naturalistic classroom scenarios has attracted increasing attention for both psychologists and educational researchers, leading to an emerging field of educational neurosciences (Lieberman, 2012; Miller, 2016). Researchers are starting to collect neurophysiological data in the classroom and the analyses of these data are supposed to reveal the underlying mechanisms for learning (Wu et al., 2016). A better understanding of how people learn will ultimately improve the learning and instruction.

The state-of-the-art wearable bio-sensing techniques are readily available for recording the neurophysiological signals from both the central nervous system (CNS) and the autonomic nervous system (ANS). Wearable electroencephalography (EEG) and wearable functional near-infrared spectroscopy (fNIRS) are the most popular signals for characterizing CNS activities. For instance, teaching outcome has been demonstrated to be correlated with fNIRS-based cortical coupling between the teacher’s and the student’s brain in dyadic settings (Holper et al., 2013; Zheng et al., 2018); simultaneous EEG recordings from groups of students have been exhibited to be associated with engagement, attention, and even their preferences for teachers (Dikker et al., 2017; Ko et al., 2017; Poulsen et al., 2017); and student-teacher synchrony in real classroom settings can reflect students’ perceived closeness to the teacher (Bevilacqua et al., 2018). Despite these recent exciting findings, the need for experts for preparing and setting up the recording devices, as well as the inevitable physical contacts between the probes/electrodes and human scalps, pose limitations for a direct application in long-term and large-scale studies, which is a necessary and important step toward providing suggestions for educational researchers and practitioners (Immordino-Yang and Gotlieb, 2017). Indeed, the experimental conditions were usually well controlled with artificial tasks and limited number of students (with size between 9 and 18 students in recent studies).

The recordings of ANS activities, however, are likely to be a more suitable candidate for monitoring students’ cognitive statuses in a more natural way. To represent ANS activities, heart rate (HR), heart rate variability, skin conductance, respiration rate, and skin temperature are the commonly used signals. These signals have been well documented to reflect critical cognitive statuses as well (Cowley et al., 2016; Wu et al., 2016). In addition, multi-dimensional representations using two or more of these signals, have been suggested to characterize more complex cognitive statuses or have better predictive powers, as compared to the single-signal unidimensional representation (Frantzidis et al., 2010; Shiota et al., 2011; Charland et al., 2015). The earliest attempt to use ANS to investigate education questions can be tracked back to Koester and Farley (1982), using both HR and skin conductance. Compared to this pioneer study, these signals can now be acquired by using wristband-like devices with high quality. Besides the maturation of the ANS recording techniques, the wristband-like devices are much cheaper and more user-friendly to wear and use than the EEG and fNIRS devices for CNS signals, with minimal influence on the students’ normal activities. Therefore, they are more suitable for studies with a high ecological validity, i.e., monitoring a larger group of students in their normal classroom environment for a longer term, e.g., for weeks, months, or years.

Whereas the majority of the ANS-based studies to date have focused on the prediction of the cognitive statuses, cognitive activities need be able to predict academic performance to be meaningful to educators. Despite the recognition of multidimensional educational outcomes, academic achievement is still considered as the primary educational outputs by both educational researchers and practitioners (Marton and Säljö, 1976). Extensive studies have shown consistent evidence that test scores serve as significant and positive predictors of future career advancement and income (e.g., Grogger and Eide, 1995; Murnane et al., 1995, 2000; Altonji and Blank, 1999; Currie and Thomas, 2001). Therefore, it is necessary for neurophysiologists and psychologists to explore further beyond cognition, to fulfill the needs by educational researchers and practitioners.

The present study is an exploratory investigation on the prediction of academic performance by neurophysiological signals. HRs and electrodermal signals (EDAs) were measured from 100 grade seven middle-school students for 2 weeks during their daily Chinese and math classes, using a customized designed wristband. These recordings were found to be an effective predictor of the students’ academic performance measured by their final exam scores. Our results provide evidences supporting the feasibility of evaluating educational outputs by wearable neurophysiological recordings.

## Materials and Methods

### Participants

All participants are from a regular middle school in Beijing. Three classes of grade 7 were selected, from which 100 students volunteered to participate in the study (mean age 12 years and 9 months, range from 12 years and 1 month to 13 years and 4 months). The study was conducted in accordance with China’s law and the Declaration of Helsinki and approved by the institutional review board (IRB) in the Department of Psychology, Tsinghua University. All the volunteer participants and their legal guardians were provided with paper-back informed consent and signed before the data collection.

### Data Collection

The EDAs and HRs were collected in Chinese and math sessions for 1 week (from Monday to Friday) in November (20th to 24th) and another week in December (11th to 15th), 2017. Students wore the wristband in everyday morning before formal sessions (8AM) begun and took off after the Chinese sessions and math sessions were done for that day. Each session lasted for 40 min. In each week, there were six Chinese sessions and six math sessions for one class of students. The total number of sessions was 72 for the three classes of students, with 36 sessions for Chinese and 36 sessions for math.

To obtain the neurophysiological signals, the participants wore customized designed wristbands on either of their hands (Psychorus, China) throughout the Chinese and math sessions. EDAs were acquired by surface electrodes with conductive gels at a sampling rate of 40 Hz. HRs were collected using the photoplethysmography (PPG) method at a sampling rate of 20 Hz. Three-axis accelerations were recorded at 20 Hz as well, but not used in the present study. The experimenters helped the students to use conductive gels and wear the devices. To minimize the disturbance on the regular teaching activity, we did not have time to check the quality of the data during preparation but performed *post hoc* artifact rejection to exclude possible low-quality data.

After each session, participants filled out a short questionnaire to report their self-assessment on the following three items: (1) the degree of knowledge mastery during this session (five options: under 30%, 30–50%, 50–70%, 70–90%, 90% above), (2) the degree of concentration during this session, and (3) the general emotional valence (negative or positive) during this session. The second and third items were rated by seven-point Likert scales. All the students were explicitly informed that their reports were just for research purposes and would never be revealed to their teachers.

Following the common practice (Marsh and Yeung, 1997; Sirin, 2005), the students’ final exam scores (in January 2018) were used to measure their academic performance.

### Data Preprocessing

Defining one student’s data collected in one session as one dataset, there were in total 2400 datasets (100 students × 24 sessions per student). As the neurophysiological recordings took place in real classrooms and the cooperation level of grade seven students were in general lower than adult participants, we performed careful visual inspections to exclude the datasets with low data qualities. Datasets were rejected if they fitted one of the following criterions: (1) no change in EDA signals for >30% of the recording time (indicating the wristband not properly worn by participants as required); (2) high frequency oscillation for >50% of the recording time (indicating no effective contact between the wristband and the skin); (3) abnormal HR values (<40 beat per minute (BPM) or >200 BPM) for >30% of the recording time. These ratios were selected empirically so as to keep a sufficient amount of data for a reliable estimation of the single session neurophysiological data. After inspection, 809 datasets from 84 students were included for further analysis.

**FIGURE 1:**
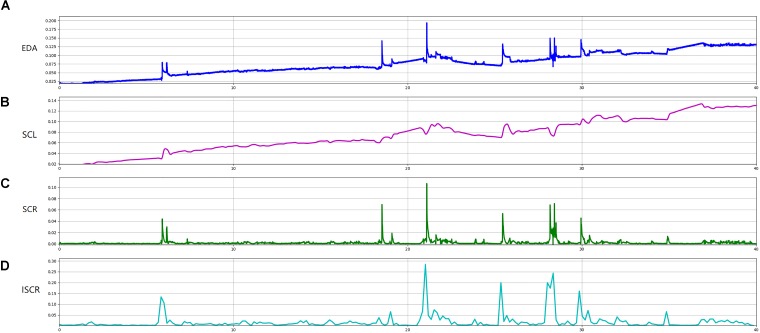
An example of a participant’s EDA curves over one classroom session. **(A)** EDA is the curve of raw data of skin conductance of one student over a sample session (40 min); **(B)** SCL is the tonic skin conductance level decomposed from the CDA method; **(C)** SCR is the transient skin conductance response decomposed from the CDA method; and **(D)** iSCR is the integral of SCR over the 10-s non-overlapping time windows.

Preprocessing of EDA signals was carried out using the LEDALAB toolbox (Benedek and Kaernbach, 2010). The raw signals were first downsampled to 10 Hz and then smoothed with an 8-point Gaussian window for noise reduction. The signals were further decomposed into the tonic skin conductance level (SCL) and the transient skin conductance response (SCR) (Boucsein, 2012), using the continuous decomposition analysis (CDA) method. Instead of defining discrete events from SCRs based on the response peaks, here the integration of SCRs (iSCR) was calculated to represent the overall SCR in a certain time period. The integration was believed to effectively capture the cumulative effect of the EDA signals, while avoiding the possible influences by the usually arbitrary decision of the thresholds for peak detection and event definition (Benedek and Kaernbach, 2010). Considering the non-stationary of the EDA signals (Son and Park, 2011), both SCL and iSCR were calculated on the basis of 10-s non-overlapping time windows for all datasets. The mean and variation of the 10-s based SCL and iSCR over each 40-min session were then extracted as the indicators of the EDA signals of each dataset. The calculation of the EDA indicators is illustrated in Figure 1.

Heart rates were calculated in a similar way using the PPG data. The mean and variation of the 10-s based averaged HRs over one session were extracted as the indicators of the HRs of the corresponding dataset.

A second-round artifact rejection procedure was then performed to check for extreme outliers, the datasets with any of the above-mentioned extracted neurophysiological indicators (i.e., mean and variation of SCL, iSCR, HR) exceeding five standard deviations of the sample mean (of all datasets) were rejected. The selection of five standard deviations was decided empirically to exclude the most extreme data while maintaining a reasonable amount of data for the following data analyses. The final number of datasets for statistical analysis was 771 from 84 students. For each of the 84 students, 1–21 datasets were available for analysis.

The academic performance, i.e., the final exam scores for Chinese and math, were standardized, respectively, over all the Grade seven students of that school (503 students, including non-participant).

### Data Analysis

Pairwise Pearson’s correlations were first calculated between all possible pairs among the neurophysiological indicators, the questionnaire reports and the corresponding academic performance (i.e., pooled final exam scores of Chinese and math). Since the neurophysiological data were clustered within each individual student (i.e., each student had data from multiple sessions), standard errors were corrected for this intra-individual correlation by using cluster-robust covariance matrix and multilevel modeling as suggested by Liang and Zeger (1986) and White (1980).

Multiple linear regression was applied to explore the relation between all the neurophysiological indicators and the academic performance as reflected by the final exam scores. Regression models were first computed for Chinese and math classes separately and then on pooled data of the two subjects. The neurophysiological indicators were used as the independent variables and the final exam scores were used as the dependent variables. Due to the high correlations among the neurophysiological indicators, principal component analysis (PCA) was used to extract the principal components to avoid collinearity, prior to the regression analysis. PCA was applied to the z-scores of the neurophysiological data and a varimax rotation was used. The Kaiser criterion (eigenvalue > 1) was employed to decide the number of factors to be retained. Similar to the correlation analysis, standard errors were corrected for intra-individual correlations by using cluster-robust covariance matrix and multilevel modeling as well.

The self-report questionnaire is the traditional method to measure students’ personal inputs in class and learning output (Schmeck et al., 1977; Pintrich and De Groot, 1990; Pekrun et al., 2002). We would like to check if physiological data could provide additional explanation to the variation of academic performance, compared with self-report data. To this end, multiple linear regression models were constructed with the session-based questionnaire reports as the independent variables and the academic performance as the dependent variable were constructed. Principal components were extracted from the questionnaire reports as well, using the PCA method. Then, the two predicted values from both the neurophysiological-based and the questionnaire-based regression models were further used together as independent variables to predict academic performance. A significant regression coefficient for any of the two predicted values would imply a unique contribution of the corresponding set of variables that cannot be explained by the other set.

Lastly, the subject-specificity of the relationship between neurophysiological data and test scores (either Chinese or math) were also investigated. Regression models were constructed by switching the dependent variables between the two subjects, while keeping the independent variables unmoved. A non-significant result by such a switch would support a subject-specific neurophysiological response pattern and a significant regression would imply a supra-subject finding. In addition to further check if this correlation is subject specific or just because of students’ consistent performance in both Chinese and math sessions, a subsample of students favoring only one subject over the other is identified by the standard that the difference between math and Chinese standardized test scores exceeding 0.5 SD. According to this standard, thirty students are identified as those favoring only subject. Similarly, the regular regression model and switched dependent variable model were applied on this subsample.

## Results

### Pairwise Correlations

The pairwise correlation results are presented in Table 1. The neurophysiological data were highly correlated: significant positive correlations were observed for the pairs of SCL mean and SCL variation (*r* = 0.584, *p* < 0.01), iSCR mean and iSCR variation (*r* = 0.901, *p* < 0.01), HR variation and iSCR mean (*r* = 0.263, *p* < 0.01)/variation (*r* = 0.240, *p* < 0.01); significant negative correlations were found for the pairs of HR variation and SCL mean (*r* = -0.091, *p* < 0.05), SCL mean with iSCR mean (*r* = -0.305, *p* < 0.01)/variation (*r* = -0.224, *p* < 0.01), and SCL variation with iSCR mean (*r* = -0.120, *p* < 0.01)/variation (*r* = -0.063, *p* < 0.01). The pooled final exam scores were positively correlated with SCL mean (*r* = 0.079, *p* < 0.05), self-reported knowledge mastery (*r* = 0.260, *p* < 0.01), and negatively correlated with iSCR mean (*r* = -0.172, *p* < 0.01)/variation (*r* = -0.154, *p* < 0.01). When considering the subject specificity, iSCR mean (*r* = -0.354, *p* < 0.001) and iSCR variation (*r* = -0.325, *p* < 0.01) were only negatively correlated with Chinese scores; and SCL mean was only positively correlated with math scores (*r* = 0.113, *p* < 0.01).

**Table 1:** The pairwise correlation matrix.

	HR mean	HR variation	SCL mean	SCL variation	iSCR mean	iSCR variation	Knowledge mastery	Attention	Emotion
HR variation	-0.047								
	(0.047)								
SCL mean	-0.087	-0.091^∗∗^							
	(0.053)	(0.037)							
SCL variation	0.005	0.005	0.584^∗∗∗^						
	(0.045)	(0.049)	(0.069)						
iSCR mean	-0.002	0.263^∗∗∗^	-0.305^∗∗∗^	-0.120^∗∗∗^					
	(0.050)	(0.056)	(0.038)	(0.029)					
iSCR variation	0.014	0.240^∗∗∗^	-0.224^∗∗∗^	-0.063^∗∗^	0.901^∗∗∗^				
	(0.051)	(0.054)	(0.039)	(0.022)	(0.046)				
Knowledge mastery	-0.122^∗^	0.060	-0.019	-0.067	-0.035	-0.027			
	(0.065)	(0.051)	(0.054)	(0.044)	(0.069)	(0.049)			
Attention	-0.075	0.059	0.008	-0.023	-0.062	-0.077	0.483^∗∗∗^		
	(0.048)	(0.055)	(0.040)	(0.028)	(0.065)	(0.052)	(0.061)		
Emotion	-0.133^∗∗^	0.099^∗^	0.007	-0.026	-0.010	0.002	0.539^∗∗∗^	0.630^∗∗∗^	
	(0.055)	(0.051)	(0.045)	(0.039)	(0.064)	(0.049)	(0.057)	(0.066)	
Final exam	-0.033	-0.051	0.079^∗∗^	0.006	-0.172^∗∗∗^	-0.154^∗∗∗^	0.260^∗∗^	0.089	0.101
	(0.073)	(0.052)	(0.036)	(0.042)	(0.061)	(0.057)	(0.101)	(0.074)	(0.067)


*^∗^*p* < 0.1, ^∗∗^*p* < 0.05, ^∗∗∗^*p* < 0.01, cluster-robust standard errors in parentheses.*

### The Relationship Between Physiological Indicators and Final Exam Scores

The loading matrix of PCA performed on the neurophysiological signals are reported in Table 2. Three neurophysiological factors (NF1, NF2, NF3) have eigenvalues larger than one and therefore retained (Kaiser’s criterion), explaining 77.7% of the total variance. According to the loading matrix, NF1 mainly represents iSCR; NF2 represents SCL and NF3 represents HR.

**Table 2:** Rotated factor loading matrix for the neurophysiological data.

Variables	NF1	NF2	NF3
HR mean	0.035	-0.011	0.964
HR variation	0.473	0.045	-0.288
SCL mean	-0.231	0.856	-0.086
SCL variation	0.011	0.908	0.049
iSCR mean	0.943	-0.137	0.014
iSCR variation	0.943	-0.055	0.038


The multiple regression results with the neurophysiological factors as independent variables and the academic performance as dependent variables are listed in Table 3. Significant prediction of the Chinese final exam scores and the pooled data by the neurophysiological data were found (*R*^2^ = 0.083 and 0.03, respectively). The major contributor in both regressions was NF1, i.e., iSCR mean and variation. Although the prediction of math scores was not significant, the regression coefficient of NF1 was also significant.

**Table 3:** Regression on final exam scores by the neurophysiological factors.

Variables	Chinese	Math	Chinese and Math
	(1)	(2)	(3)
NF1	-0.291^∗∗∗^(0.083)	-0.082^∗^(0.044)	-0.138^∗∗∗^(0.046)
NF2	0.000(0.032)	0.054(0.033)	0.023(0.027)
NF3	0.024(0.081)	-0.060(0.060)	-0.029(0.061)

Sample size	345	426	771
*F*-value	4.66	1.90	3.03
*P*-value	0.005	0.137	0.034
*R*^2^	0.083	0.020	0.030


*^∗^*p* < 0.1, ^∗∗^*p* < 0.05, ^∗∗∗^*p* < 0.01, cluster-robust standard errors in parentheses.*

### Comparison Between the Neurophysiological Data and Self-Report Data

A PCA was first run on self-reported data to keep the analysis consistent, and one self-report factor (SF1) was retained (Table 4), explaining 70.1% of the total variance. The regression results of self-report factor on final scores were presented in Table 5. The self-reports on the pooled data significantly predicted the final exam scores (*R*^2^ = 0.031, *p* = 0.054) but the subject-specific models showed different results: While the self-reports on math sessions could marginally significantly predict the final exam scores (*R*^2^ = 0.033, *p* = 0.054), while the self-reports on Chinese sessions failed to do so (*R*^2^ = 0.026, *p* = 0.182).

**Table 4:** Loading matrix on self-report variables.

Variables	SF1
Q1-Mastery of knowledge	0.795
Q2-Attention	0.845
Q3-Emotional valence	0.871


**Table 5:** Analysis on self-report to final exam scores.

	Chinese	Math	Chinese and Math
	(1)	(2)	(3)
SF1	0.137(0.104)	0.149^∗^(0.076)	0.146^∗∗^(0.066)

Sample size	345	426	771
*F*-value	1.82	3.82	4.84
*P*-value	0.182	0.054	0.031
*R*^2^	0.026	0.033	0.031


*^∗^*p* < 0.1, ^∗∗^*p* < 0.05, ^∗∗∗^*p* < 0.01, cluster-robust standard errors in parentheses.*

The results of the regression models with both the neurophysiological data and self-report data are summarized in Table 6. The pooled model showed significant contributions by both these two types of data, as reflected by the significant regression coefficients. Similar findings were observed for math classes as well, but the regression coefficient of the self-report data during the Chinese classes failed to reach a significant level. Increases of the regression *R*^2^ values were also observed, as compared to the single-indicator based models. The largest *R*^2^ value was obtained for the Chinese-class based model, reaching 0.095.

**Table 6:** Regression on both self-report and neurophysiological data.

	Chinese	Math	Chinese and Math
	(1)	(2)	(3)
Self-report	0.895(0.638)	0.988^∗^(0.510)	0.951^∗∗^(0.430)
Neurophysiological	1.829^∗∗∗^(0.621)	0.615^∗∗^(0.291)	0.949^∗∗∗^(0.325)

Sample size	345	426	771
*F*-value	5.34	2.93	5.81
*P*-value	0.007	0.060	0.004
*R*^2^	0.095	0.046	0.057


*^∗^*p* < 0.1, ^∗∗^*p* < 0.05, ^∗∗∗^*p* < 0.01, cluster-robust standard errors in parentheses.*

### Subject-Specificity

The regression analysis results with the subject scores switched are shown in Table 7. Based on the neurophysiological data, all regression models failed to reach a significant level (although marginal significant for Chinese). Nevertheless, the coefficients for NF1 remained to be significant for Chinese and pooled data.

**Table 7:** Regression on subject-switched data.

	Chinese	Math	Chinese and Math
	(1)	(2)	(3)
NF1	-0.221^∗∗^(0.100)	-0.090(0.056)	-0.126^∗∗^(0.054)
NF2	-0.032(0.033)	0.060^∗^(0.034)	0.010(0.030)
NF3	-0.027(0.103)	0.030(0.054)	0.006(0.061)

Sample size	345	426	771
*F*-value	2.65	1.15	2.22
*P*-value	0.055	0.334	0.092
*R*^2^	0.043	0.020	0.022


*^∗^*p* < 0.1, ^∗∗^*p* < 0.05, ^∗∗∗^*p* < 0.01, cluster-robust standard errors in parentheses.*

A further exploration with a subsample focusing on students favoring one subject only are listed in Table 8. Among all the regression models, the ones with mismatched exam scores revealed non-significant results and the ones with matched scores were toward significance. Notably, the regression for Chinese scores reached a *R*^2^ value as high as 0.162 (*p* < 0.001).

**Table 8:** Subject specific check on one-subject preferred students.

	Chinese class		Math class		Chinese and Math	
			
	Chinese score	Math score	Math score	Chinese score	Corresponding score	Switched score
	(1)	(2)	(3)	(4)	(5)	(6)
NF1	-0.328^∗∗∗^(0.052)	-0.239(0.140)	-0.023(0.075)	-0.028(0.106)	-0.109^∗^(0.062)	-0.099(0.088)
NF2	-0.020(0.087)	-0.115(0.090)	0.048(0.071)	0.092(0.069)	-0.002(0.060)	-0.012(0.076)
NF3	-0.275(0.211)	-0.323(0.348)	-0.364^∗^(0.206)	-0.171(0.136)	-0.306(0.198)	-0.224(0.201)

Sample size	108	108	141	141	249	249
*F*-value	13.97	1.17	2.43	1.30	2.59	0.79
*P*-value	0.000	0.340	0.087	0.294	0.071	0.511
*R*^2^	0.162	0.096	0.101	0.035	0.086	0.046


*^∗^*p* < 0.1, ^∗∗^*p* < 0.05, ^∗∗∗^*p* < 0.01, cluster-robust standard errors in parentheses. A subsample was selected who are identified as those favoring one subject only. The column (1), (3), and (5) show the results of regression on corresponding final Chinese, math and total scores separately and the column (2), (4), and (6) present the results of regression on subject-switched final exam scores. In column (2), Chinese sessions data were applied to predict math scores, and in column (4), math sessions data were used to predict Chinese scores.*

## Discussion

In the present study, we explored the predictability of the neurophysiological recordings in the classroom for academic performance in a middle-school cohort. The wrist recorded EDAs were found to be an effective indicator of the students’ academic performance, with better results for the Chinese classes than the math classes. Compared to the session-based self-reports, these neurophysiological signals were shown to provide additional information. Furthermore, the predication of the neurophysiological signals was revealed to be subject specific. Taken together, our results provide preliminary evidences toward the application of wearable neurophysiological recordings for the evaluation of educational outputs.

Among all the six types of neurophysiological signals, SCL mean, iSCR mean, and iSCR variation were shown to be significantly correlated with the students’ academic performance. As the tonic skin conductance level (SCL mean) has been suggested to reflect the general arousal or activation level (Bortoletto et al., 2011), the positive correlation between SCL mean and the final exam scores indicated that the students with better academic performance were more activated when attending the classes. The negative correlation between the final exam scores and the mean of the transient skin conductance response (iSCR mean), could be attributed to an overall reduced response magnitude, or a reduced number of transient responses to the possible classroom events. The negative effect of the variation of the transient skin conductance response over all 10-s segments within one session (iSCR variation) on exam scores, further implied that the stability of the students’ neurophysiological responses over time could be critical for their academic performance.

Chinese and math represent two kinds of competence. In Chinese and math classes, students need to use very different emotional and cognitive strategies in order to learn well. Accordingly, the regression models were found to be specific for the Chinese and the math classes: the regression models with the switched final scores reported non-significant results, and significant regression results were achieved for the Chinese classes, as well as the pooled data. This finding suggests at least partly distinct neurophysiological activity patterns during the two types of classes. Indeed, iSCR performs better in predicting Chinese score than in explaining math score; while SCL mean only positively correlates with math core. These two results indicate that high math achievers have higher activation and more stable response; while high Chinese achievers only need even more stable responses in the classroom. More stable response may due to lower effort or better emotion regulation (Shi et al., 2007; Nourbakhsh et al., 2012; Christopoulos et al., 2016). It might due to the fact that math class is more challenging and more structured, therefore the activation level is higher but the needs for emotion regulation is relatively lower, compared with Chinese class, in which the content is not so challenging and not so clearly structured (e.g., Chinese class has more group discussions, more free writing time, and even performance and presentations), and therefore needs more emotion regulation to study well.

Importantly, the regression analysis with both the neurophysiological data and the self-report data revealed independent contributions from the neurophysiological data in both Chinese and math classes. In other words, students’ neurophysiological data provided additional information about their final exam scores that could not be explained by self-reports. Such observation provides strong support for the necessity of recording neurophysiological data in real classroom environment, in line with the general opinions on physiological computing (Rutherford, 2010; Jacucci et al., 2015; Cowley et al., 2016). By combining the two types of information, a better and more complete understanding of the students’ learning process is expected to be achieved.

While the link between human neurophysiological signals and cognitive functions has been well established, our study demonstrates a direct link toward academic performance. As wearable bio-sensing techniques are capable of continuously recording students’ neurophysiological signals without interrupting normal classroom activities, our results suggest wearable neurophysiological recording devices as a useful tool for educational research and practices. With the emerging trend along this promising new direction, there is a strong call for further studies that integrate neuroscience and educational research with high ecological validity.

Notably, whereas ideally it is expected to record data from all sessions in this semester for the prediction of the academic performances, the data were measured 2 weeks with an interval of approximately a month due to feasibility issues, mainly about possible disturbances on the regular school activities. Therefore, the found correlation between the recorded neurophysiological signals and the academic performances might be explained by a relatively stable status of the students over all sessions. Alternatively, it might also be possible that the neurophysiological activities reflected a general but subject-specific cognitive capability. Nevertheless, it remains to be elucidated whether more recording data could provide a better prediction of the academic performances.

## Author Contributions

YZ and DZ lead this project. YZ, FQ, and DZ did the data analysis and drafted the paper. BL did the data processing. XQ and YYZ coordinated the data collection.

## Conflict of Interest Statement

The authors declare that the research was conducted in the absence of any commercial or financial relationships that could be construed as a potential conflict of interest.

## References

[B1] Altonji J. G. Blank R. M. (1999). Race and gender in the labor market. *Handb. Labor Econ.* 3 3143–3259. 10.1016/S1573-4463(99)30039-0

[B2] Benedek M. Kaernbach C. (2010). A continuous measure of phasic electrodermal activity. *J. Neurosci. Methods* 190 80–91. 10.1016/j.jneumeth.2010.04.028 20451556 PMC2892750

[B3] Bevilacqua D. Davidesco I. Wan L. Oostrik M. Chaloner K. Rowland J. (2018). Brain-to-Brain synchrony and learning outcomes vary by student–teacher dynamics: evidence from a real-world classroom electroencephalography study. *J. Cogn. Neurosci.* 59 1–11. 10.1162/jocn_a_01274 29708820

[B4] Bortoletto M. Lemonis M. J. Cunnington R. (2011). The role of arousal in the preparation for voluntary movement. *Biol. Psychol.* 87 372–378. 10.1016/j.biopsycho.2011.04.008 21557986

[B5] Boucsein W. (2012). *Electrodermal Activity.* Berlin: Springer Science & Business Media 10.1007/978-1-4614-1126-0

[B6] Charland P. Léger P. M. Sénécal S. Courtemanche F. Mercier J. Skelling Y. (2015). Assessing the multiple dimensions of engagement to characterize learning: a neurophysiological perspective. *J. Vis. Exp.* 101:e52627. 10.3791/52627 26167712 PMC4544848

[B7] Christopoulos G. I. Uy M. A. Yap W. J. (2016). The body and the brain: measuring skin conductance responses to understand the emotional experience. *Organ. Res. Methods* 10.1177/1094428116681073

[B8] Cowley B. Filetti M. Lukander K. Torniainen J. Henelius A. Ahonen L. (2016). The psychophysiology primer: a guide to methods and a broad review with a focus on human–computer interaction. *Found. Trends Hum. Comp. Interact.* 9 151–308. 10.1561/1100000065

[B9] Currie J. Thomas D. (2001). “Early test scores, school quality and SES: longrun effects on wage and employment outcomes,” in *Worker Wellbeing in a Changing Labor Market* (Bingley: Emerald Group Publishing Limited), 103–132. 10.1016/S0147-9121(01)20039-9

[B10] Dikker S. Wan L. Davidesco I. Kaggen L. Oostrik M. McClintock J. (2017). Brain-to-Brain synchrony tracks real-world dynamic group interactions in the classroom. *Curr. Biol.* 27 1375–1380. 10.1016/j.cub.2017.04.002 28457867

[B11] Driver J. (2001). A selective review of selective attention research from the past century. *Br. J. Psychol.* 92 53–78. 10.1348/000712601162103 11256770

[B12] Frantzidis C. A. Bratsas C. Papadelis C. L. Konstantinidis E. Pappas C. Bamidis P. D. (2010). Toward emotion aware computing: an integrated approach using multichannel neurophysiological recordings and affective visual stimuli. *IEEE Trans. Inform. Technol. Biomed.* 14 589–597. 10.1109/TITB.2010.2041553 20172835

[B13] Grogger J. Eide E. (1995). Changes in college skills and the rise in the college wage premium. *J. Hum. Resour.* 30 280–310. 10.2307/146120

[B14] Holper L. Goldin A. P. Shalóm D. E. Battro A. M. Wolf M. Sigman M. (2013). The teaching and the learning brain: a cortical hemodynamic marker of teacher–student interactions in the Socratic dialog. *Int. J. Educ. Res.* 59 1–10. 10.1016/j.ijer.2013.02.002

[B15] Immordino-Yang M. H. Gotlieb R. (2017). Embodied brains, social minds, cultural meaning: integrating neuroscientific and educational research on social-affective development. *Am. Educ. Res. J.* 54 344S–367S. 10.3102/0002831216669780

[B16] Jacucci G. Fairclough S. Solovey E. T. (2015). Physiological computing. *Computer* 48 12–16. 10.1109/MC.2015.291

[B17] Ko L.-W. Komarov O. Hairston W. D. Jung T.-P. Lin C.-T. (2017). Sustained attention in real classroom settings: an EEG study. *Front. Hum. Neurosci.* 11:440. 10.3389/fnhum.2017.00388 28824396 PMC5534477

[B18] Koester L. S. Farley F. H. (1982). Psychophysiological characteristics and school performance of children in open and traditional classrooms. *J. Educ. Psychol.* 74 254–263. 10.1037/0022-0663.74.2.254 7076971

[B19] Li X. Zhang P. Song D. Hou Y. (2015). Recognizing emotions based on multimodal neurophysiological signals. *Adv. Comput. Psychophysiol.* 28–30.

[B20] Liang K. Y. Zeger S. L. (1986). Longitudinal data analysis using generalized linear models. *Biometrika* 73 13–22. 10.1093/biomet/73.1.13

[B21] Lieberman M. D. (2012). Education and the social brain. *Trends Neurosci. Educ.* 1 3–9. 10.1016/j.tine.2012.07.003

[B22] Marsh H. W. Yeung A. S. (1997). Causal effects of academic self-concept on academic achievement: structural equation models of longitudinal data. *J. Educ. Psychol.* 89 41–54. 10.1037/0022-0663.89.1.41

[B23] Marton F. Säljö R. (1976). On qualitative differences in learning: I—Outcome and process. *Br. J. Educ. Psychol.* 46 4–11. 10.1111/j.2044-8279.1976.tb02980.x

[B24] Miller R. (2016). Neuroeducation: integrating brain-based psychoeducation into clinical practice. *J. Ment. Health Couns.* 38 103–115. 10.17744/mehc.38.2.02

[B25] Murnane R. J. Willett J. B. Duhaldeborde Y. Tyler J. H. (2000). How important are the cognitive skills of teenagers in predicting subsequent earnings? *J. Policy Anal. Manage.* 19 547–568. 10.1002/1520-6688(200023

[B26] Murnane R. J. Willett J. B. Levy F. (1995). *The Growing Importance of Cognitive Skills in Wage Determination (No. w5076).* Cambridge, MA: National Bureau of Economic Research 10.3386/w5076

[B27] Nourbakhsh N. Wang Y. Chen F. Calvo R. A. (2012). “Using galvanic skin response for cognitive load measurement in arithmetic and reading tasks,” in *Proceedings of the 24th Australian Computer-Human Interaction Conference* (New York, NY: ACM), 420–423. 10.1145/2414536.2414602

[B28] Pekrun R. Goetz T. Titz W. Perry R. P. (2002). Academic emotions in students’ self-regulated learning and achievement: a program of qualitative and quantitative research. *Educ. Psychol.* 37 91–105. 10.1207/S15326985EP3702_4

[B29] Picard R. W. Vyzas E. Healey J. (2001). Toward machine emotional intelligence: analysis of affective physiological state. *IEEE Trans. Pattern Anal. Mach. Intell.* 23 1175–1191. 10.1109/34.954607

[B30] Pintrich P. R. De Groot E. V. (1990). Motivational and self-regulated learning components of classroom academic performance. *J. Educ. Psychol.* 82:33 10.1037/0022-0663.82.1.33

[B31] Poulsen A. T. Kamronn S. Dmochowski J. Parra L. C. Hansen L. K. (2017). EEG in the classroom: synchronised neural recordings during video presentation. *Sci. Rep.* 7:43916. 10.1038/srep43916 28266588 PMC5339684

[B32] Rutherford J. J. (2010). Wearable technology. *IEEE Eng. Med. Biol. Mag.* 29 19–24. 10.1109/MEMB.2010.936550 20659854

[B33] Schmeck R. R. Ribich F. Ramanaiah N. (1977). Development of a self-report inventory for assessing individual differences in learning processes. *Appl. Psychol. Meas.* 1 413–431. 10.1177/014662167700100310

[B34] Shi Y. Ruiz N. Taib R. Choi E. Chen F. (2007). “Galvanic skin response (GSR) as an index of cognitive load,” in *CHI’07 Extended Abstracts on Human Factors in Computing Systems* (New York, NY: ACM), 2651–2656. 10.1145/1240866.1241057

[B35] Shiota M. N. Neufeld S. L. Yeung W. H. Moser S. E. Perea E. F. (2011). Feeling good: autonomic nervous system responding in five positive emotions. *Emotion* 11 1368–1378. 10.1037/a0024278 22142210

[B36] Sirin S. R. (2005). Socioeconomic status and academic achievement: a meta-analytic review of research. *Rev. Educ. Res.* 75 417–453. 10.3102/00346543075003417

[B37] Son J. Park M. (2011). “Estimating cognitive load complexity using performance and physiological data in a driving simulator,” in *Adjunct Proceedings of the Automotive User Interfaces and Interactive Vehicular Applications Conference*, New York, NY.

[B38] White H. (1980). A heteroskedasticity-consistent covariance matrix estimator and a direct test for heteroskedasticity. *Econometrica* 48 817–838. 10.2307/1912934

[B39] Wu C. H. Huang Y. M. Hwang J. P. (2016). Review of affective computing in education/learning: trends and challenges. *Br. J. Educ. Technol.* 47 1304–1323. 10.1111/bjet.12324

[B40] Zheng L. Chen C. Liu W. Long Y. Zhao H. Bai X. (2018). Enhancement of teaching outcome through neural prediction of the students’ knowledge state. *Hum. Brain Mapp.* 7 3046–3057. 10.1002/hbm.24059 29575392 PMC6866636

